# Cullin 3 Recognition Is Not a Universal Property among KCTD Proteins

**DOI:** 10.1371/journal.pone.0126808

**Published:** 2015-05-14

**Authors:** Giovanni Smaldone, Luciano Pirone, Nicole Balasco, Sonia Di Gaetano, Emilia Maria Pedone, Luigi Vitagliano

**Affiliations:** 1 Institute of Biostructures and Bioimaging, C.N.R., 80134, Napoli, Italy; 2 Institute of Crystallography, C.N.R., 70126, Bari, Italy; 3 Second University of Napoli, 81100, Caserta, Italy; George Washington University, UNITED STATES

## Abstract

Cullin 3 (Cul3) recognition by BTB domains is a key process in protein ubiquitination. Among Cul3 binders, a great attention is currently devoted to KCTD proteins, which are implicated in fundamental biological processes. On the basis of the high similarity of BTB domains of these proteins, it has been suggested that the ability to bind Cul3 could be a general property among all KCTDs. In order to gain new insights into KCTD functionality, we here evaluated and/or quantified the binding of Cul3 to the BTB of KCTD proteins, which are known to be involved either in cullin-independent (KCTD12 and KCTD15) or in cullin-mediated (KCTD6 and KCTD11) activities. Our data indicate that KCTD6^BTB^ and KCTD11^BTB^ bind Cul3 with high affinity forming stable complexes with 4:4 stoichiometries. Conversely, KCTD12^BTB^ and KCTD15^BTB^ do not interact with Cul3, despite the high level of sequence identity with the BTB domains of cullin binding KCTDs. Intriguingly, comparative sequence analyses indicate that the capability of KCTD proteins to recognize Cul3 has been lost more than once in distinct events along the evolution. Present findings also provide interesting clues on the structural determinants of Cul3-KCTD recognition. Indeed, the characterization of a chimeric variant of KCTD11 demonstrates that the swapping of α2β3 loop between KCTD11^BTB^ and KCTD12^BTB^ is sufficient to abolish the ability of KCTD11^BTB^ to bind Cul3. Finally, present findings, along with previous literature data, provide a virtually complete coverage of Cul3 binding ability of the members of the entire KCTD family.

## Introduction

The BTB (Bric-à-brac, Tramtrack, Broad complex) domain is a widespread structural module that is frequently involved in both self and hetero association [1]. Proteins containing BTB domains display large extreme variabilities both in terms of function and structure. In recent years, it has been shown that members of a small family of BTB containing proteins denoted as KCTD (proteins containing a K+ channel tetramerization domain) play crucial roles in fundamental biological processes [2,3,4]. Indeed, these proteins are involved in distinct physiological processes such as protein ubiquitination and degradation [5,6,7,8], suppression of proliferation or transcription [4,9], human genetic disease risk [10], sleep homeostasis [11], regulation of G-protein coupled receptors [12,13] and others. It has been pointed out that the ability of these relatively similar proteins to be implicated in diverse biological function is a topic that deserves a particular attention [2,3]. Despite their involvement in these fundamental biological processes, the biochemical roles of these proteins are often unknown. Several members of the family are involved in protein ubiquitination as part of the CRL (Cullin RING Ligase) E3 ligases. In the last decade, direct or indirect evidences of the ability of these proteins to bind simultaneously cullin 3 (Cul3) and the substrate to be ubiquitinated have been reported [6,8,14]. Cullin-independent functions of KCTD proteins include binding and regulation of GABA (g-aminobutyric acid) receptors (KCTD8, KCTD12 and KCTD16) [12,13] and inhibition of AP-2 function (KCTD15) [15].

The analysis of the molecular organization of these proteins indicates that KCTDs are characterized by a modular organization that couples BTB domains, always located at the N-terminus, to C-terminal regions that are frequently unrelated. Comparative analyses of KCTD sequences have highlighted the possibility to cluster KCTD human paralogs in seven clades (denoted with A to G letters), each composed of proteins involved in similar biological processes [2]. Notably, BTB domains of KCTDs share a significant level of sequence identity, even in cases in which the proteins are endowed with different functions. This observation opens the question whether KCTD proteins involved in cullin-independent function still retain the ability to bind Cul3. Moreover, it is currently believed that Cul3 binding function is fairly evenly spread through all branches of the KCTD family tree [2].

In this framework, we have here checked the interaction of Cul3 with two proteins (KCTD12 and KCTD15) which play crucial roles in non-cullin dependent functions. In particular, KCTD12, and the other members of the Clade F (KCTD8 and KCTD16), are integral components and modulators of the GABA(B) receptor [12,13]. KCTD12 induces fast and pronounced desensitization of the K+ current response of the channel, which results from a dual interaction with the G protein [16,17]. Nevertheless, in a systematic quantitative proteomic analysis of the CRL network, KCTD12 has been identified as a potential Cul3 interactor [18]. On the other hand, KCTD15 has an important role in regulating the neural crest domain in vertebrate embryos [15]. In particular, KCTD15 inhibits neural crest induction by the interaction with the transcription factor AP-2α activation domain, thus blocking its activity. Further, the KCTD15 gene is associated with obesity although the molecular basis for this link is currently unknown. Moreover, since a quantification of the binding of KCTD proteins to Cul3 has been reported only for the pentameric KCTD5 [19], here we also evaluated the cullin binding to two well-characterized KCTDs such as KCTD6 and KCTD11, which have been reported to bind Cul3. In particular, it has been reported that KCTD11, a negative regulator of the Hedgehog pathway [20,21], is involved in the ubiquitination and degradation of HDAC1 [6] whereas KCTD6 is also substrate adaptor for Cul3 that regulates protein levels of the muscle small ankyrin-1 isoform 5 (sAnk1.5) [22] and that, in combination with KCTD11, also participates in ubiquitination and degradation of HDAC1 [7].

## Materials and Methods

### Expression and purification of the proteins

The BTB domains of KCTD6 (KCTD6^BTB^), KCTD11 (KCTD11^BTB^), and KCTD12 (KCTD12^BTB^) were expressed in *E*. *coli* BL21(DE3) and purified by following previously reported protocols. In particular, KCTD11^BTB^, which comprises residues 15–116 of the long form of the protein (isoform 2 of the UniProt Code Q693B1-2), was obtained by following the procedure reported in Correale et al. [5]. KCTD6^BTB^, which includes the residues 10–110 of the protein (UniProt Code Q8NC69) was expressed and purified according to the protocol developed by Pirone et al. [23]. Finally, KCTD12^BTB^, which includes residues 27–131 of the protein (UniProt Code Q96CX2) was obtained according to the procedure reported by Correale et al [24] and by Errington et al. [25].

The chimeric BTB domain denoted as CHIM11/12^BTB^ was generated by replacing the residues 42–63 of KCTD11^BTB^ with the residues 61–80 of KCTD12^BTB^. Cloning, expression and purification were performed by following the procedure used for KCTD11 [5].

The BTB domain of KCTD15 (KCTD15^BTB^), which comprises residues 61–157 of the protein (UniProt Code Q96SI1-1) was expressed and characterized in the present investigations.

BTB15Fw (5’-GGAATTCCCATATGGCACCTGTGCACATCGATGT-3’) and BTB15Rv (5’-CGGAATTCTTAGGCCCGGCTGCGGCGC-3’) primers, containing *Nde*I and *EcoR*I restriction sites, were used to amplify KCTD15^BTB^, using human KCTD15^BTB^ cDNA as template (Open Biosystems). The PCR product was cloned into pET28b+ expression vector (EMBL, Grenoble) with an N-terminal polyHis tag. *E*. *coli* BL21(DE3) pLys-S strain was co-transformed with the recombinant plasmid for KCTD15^BTB^ and with pREP4 GroESL plasmid. Induction was performed at 18°C for 16 h by the addition of 0.5 mM IPTG. Cells were then harvested and the proteins were isolated by sonicating cell pellets resuspended in 50 ml lysis buffer (20 mM NaP pH 7.5, 500 mM NaCl) in the presence of an EDTA free protease inhibitor cocktail (Roche Diagnostics). The crude cell extracts were cleared by centrifugation at 18 000 rpm, and the supernatants were loaded onto a 5 ml Ni-NTA column connected to AKTA FPLC system (GE Healthcare, Milan, Italy) equilibrated with binding buffer (20 mM NaP pH 7.5, 500 mM NaCl). After washing with 10 volumes of binding buffer, the protein was eluted by applying a step gradient of imidazole (40–250–500 mM). A Superdex200 10/30 (GE Healthcare) column (20 mM NaP, 200 mM NaCl, 2 mM DTT, pH 7.5) was used in the final purification step.

Cul3^NTD^, which corresponds to the N-terminal region (residues 20–381) of cullin 3, was expressed and purified according to the procedure reported by Balasco et al. [19]. The homogeneity of the proteins was checked by SDS-PAGE (data not shown).

### Biophysical characterizations

The correct folding of all proteins was assessed by Far-UV CD spectroscopy (S1 Fig). The spectra were recorded on a Jasco J-710 spectropolarimeter equipped with a Peltier thermostatic cell holder (Jasco, model PTC-343) using 1 mm path length cell at 190–260 nm. Recordings were carried out at a temperature of 20°C under constant N_2_ flow using the following parameters: scanning speed of 20 nm min^−1^, band width of 2 nm, and response time of 4 s.

The oligomeric state of KCTD15^BTB^ and CHIM11/12^BTB^ was evaluated using a SEC-LS system consisting of a semi-preparative size-exclusion chromatography column (Superdex200 10/30, GE Healthcare) coupled to a light scattering detector (miniDAWN TREOS-Wyatt Technology) and a differential refractive index detector (Shodex RI-101). The scattering data were collected, recorded, and processed by using the Astra (5.3.4 version, Wyatt Technology Corporation) software.

### Gel filtration analyses

KCTD6^BTB^, KCTD11^BTB^, KCTD12^BTB^, KCTD15^BTB^ and CHIM11/12^BTB^ were mixed with Cul3^NTD^ at 4°C in a buffer containing 20 mM NaP, 200 mM NaCl, and 2 mM DTT (pH 7.5). The samples were loaded on a Superdex 200 10/30 (GE Healthcare) column previously equilibrated with the buffer used for the incubation.

### Isothermal titration calorimetry

Isothermal titration calorimetry (ITC) studies were performed at 22°C with an ITC200 calorimeter (MicroCal/GE Healthcare,Milan, Italy). KCTD6^BTB^, KCTD11^BTB^, KCTD12^BTB^, and CHIM11/12^BTB^ (55 μM—expressed as tetramer) were titrated into a solution of Cul3^NTD^ (25 μM). Due to the tendency of KCTD15^BTB^ to aggregate when stored at high concentration, for this protein the ITC experiment was reversed by titrating Cul3^NTD^ (250 μM) with KCTD15^BTB^ (7 μM—expressed as tetramer). Preliminarily, all proteins were extensively dialyzed in a buffer containing sodium phosphate NaP 20 mM, 200 mM NaCl, and 2 mM DTT (pH 7.5). Fitting of data to a single-binding site model was conducted with the Origin software as supplied by GE Healthcare. ITC runs were repeated twice to evaluate the reproducibility of the results.

### Molecular dynamics: model and protocols

In order to gain insights into the structural determinants of cullin recognition by KCTDs, molecular dynamics (MD) simulations were performed on the BTB domain of KCTD11 and CHIM11/12. In both cases, the homology model of KCTD11^BTB^, generated as described in De Paola et al. [26], was used as a starting model. In CHIM11/12^BTB^ the region corresponding to the α2β3 loop (residues 42–63) of KCTD11^BTB^ was replaced with the corresponding residues of KCTD12 (residues 61–80). The 3D model of the α2β3 loop in CHIM11/12^BTB^ was generated by using the Swiss Model server (http://swissmodel.expasy.org/). The energy of the CHIM11/12^BTB^ model was minimized by using GROMACS software package 4.5.5 [27].

MD simulations were performed using the GROMACS software package 4.5.5. The force field OPLS-AA and the TIP4P water model were used. The models were immersed in cubic boxes whose dimensions were 8.127 × 8.127 × 8.127 nm^3^ (number of water molecules 62668) and 8.474 × 8.474 × 8.474 nm^3^ (number of water molecules 72200) for KCTD11^BTB^ and CHIM11/12^BTB^, respectively. Four Cl^−^ charge-balancing counterions were added to neutralize the overall positive charge of the CHIM11/12^BTB^ system. No ion was added for KCTD11^BTB^ since this system is neutral. The simulations were run with periodic boundary conditions. The system was equilibrated in two steps. First, the temperature of the systems was stabilized at 300 K (NVT ensemble). Equilibration of pressure at 1 atm was then conducted under an NPT ensemble. Before starting the MD simulations, energies were minimized by fixing the protein atoms and then without restraints. The system temperature was brought to 300 K in a step-wise mode. In particular, 100 ps MD runs were carried out at 50, 100, 150, 200, 250, and 300 K. The timescale were 100 ns with a time step of 0.002 ps. Bond lengths were constrained by using the LINCS procedure. Lennard-Jones interactions were calculated with a cutoff of 10 Å. Electrostatic interactions were treated using the Particle Mesh Ewald (PME) method with a grid spacing of 0.12 nm.

The collective motions of the proteins in the MD simulations were examined by Essential Dynamics, ED [28]. The covariance matrix of the coordinate fluctuations was diagonalized to obtain eigenvectors and eigenvalues. The convergence of each individual simulation in the essential space was checked by calculating the root mean square inner product (RMSIP) between two halves of the equilibrated trajectory [28,29]. The RMSIP between the first 10 eigenvectors is defined as

$$\sqrt{\frac{1}{10}\sum_{i=1}^{10}\sum_{j=1}^{10}{\left(\eta_{i}^{a}\eta_{j}^{b}\right)}^{2}}$$

where $\eta_{i}^{a}$ and $\eta_{j}^{b}$ are the *i* ^*th*^ and *j* ^*th*^ eigenvectors from the first and second half of the equilibrated trajectory, respectively.

GROMACS routines and the program VMD [30] were used in order to assess the quality of the simulations. H-bond interactions were checked by using GROMACS utilities which consider hydrogen-donor-acceptor angles <30° and donor-acceptor distances <3.5 Å.

## Results

### Biophysical characterization of the BTB domains

**Fig 1:**
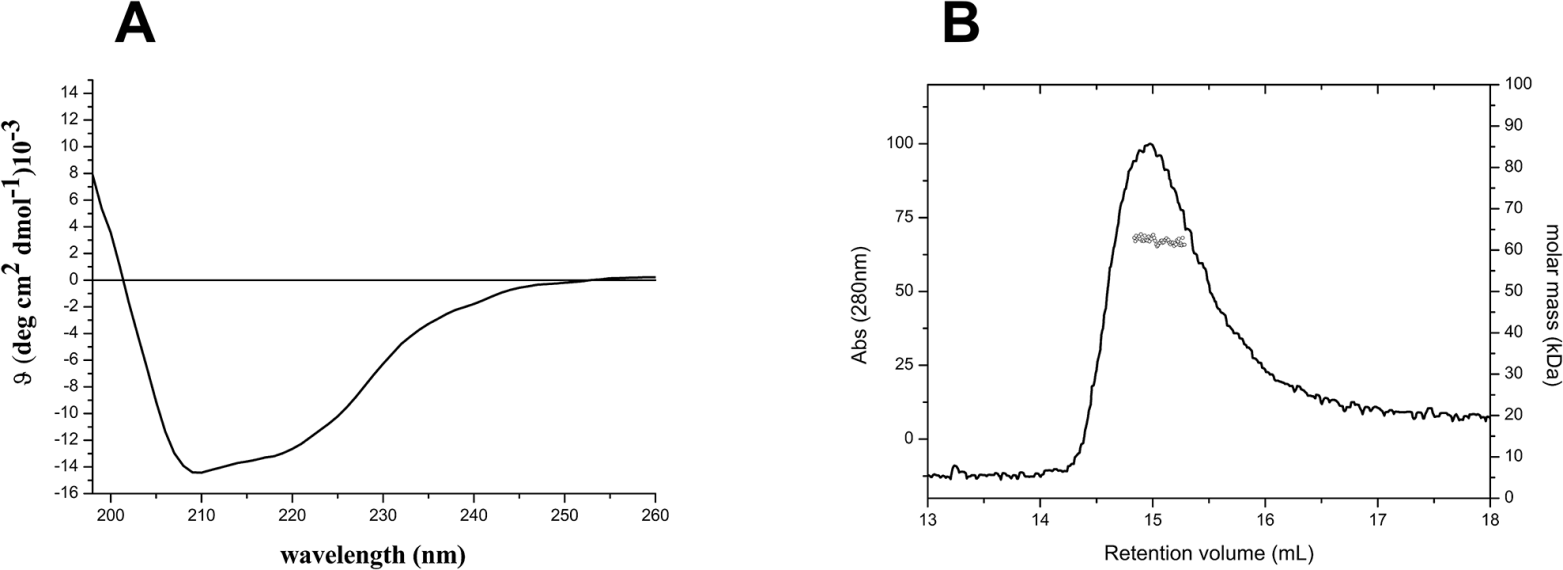
Biophysical characterization of KCTD15^BTB^ conducted by Far-UV CD spectroscopy (A) and by light scattering (B). The experiments were carried out in a 20mM sodium phosphate buffer (pH 7.5) containing 2 mM DTT.

The analysis of the interactions between KCTDs and Cul3 was performed by selecting members of the family (KCTD6, KCTD11, KCTD12, and KCTD15) that are involved in distinct biological functions. A preliminary characterization of the BTB domains of these proteins, expressed and purified in large and homogenous amounts in *E*.*coli*, was conducted to assess their folding and oligomeric state. The circular dichroism spectra of KCTD6^BTB^, KCTD11^BTB^, and KCTD12^BTB^ are identical to those reported in previous studies [5,23,24] and indicate that they adopt a α/β fold (S1 Fig). The light scattering experiments confirm that all these proteins assumed a tetrameric organization, in line with previous analyses [5,23,24] (data not shown). The BTB domain of KCTD15, whose biophysical characterization had not been previously reported, also assumes a α/β fold (Fig 1A). The molecular weight (Mw) of KCTD15^BTB^ (47.1 ± 0.4 kDa) as derived from the light scattering analysis is in line with the expected Mw of the tetramer (47.5 kDa), thus suggesting a tetrameric association of the protein (Fig 1B). This finding is somewhat expected since, in evolutionary terms, this BTB domain is close to the clade of the tetrameric KCTDs such as KCTD6, KCTD11, and KCTD12 [2]. Moreover, it is likely that KCTD1, which is closely related to KCTD15 (81% sequence identity), is also tetrameric.

### Detection and quantification of Cul3-KCTDs binding by gel filtration and ITC experiments

**Fig 2:**
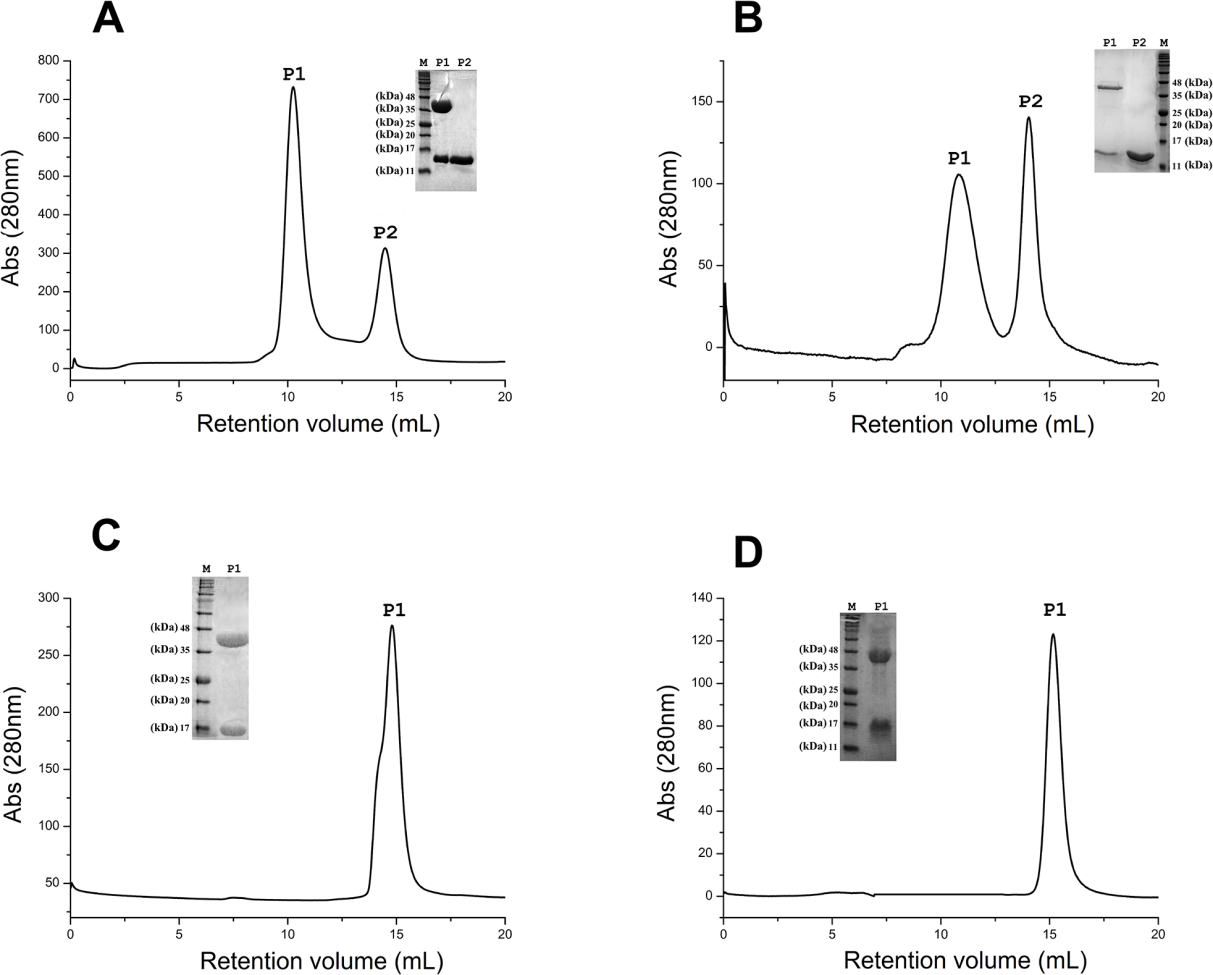
Detection of Cul3-KCTDs binding by gel filtration. Gel filtration elution profiles of KCTD6^**BTB**^ (A), KCTD11^**BTB**^ (B), KCTD12^**BTB**^ (C), and KCTD15^**BTB**^(D) after their mixing with Cul3^**NTD**^. The insets report the SDS-PAGE analysis of the peaks.

An initial analysis of the binding of these BTB domains to Cul3 was assessed by gel filtration, which provides estimates of the molecular masses of the proteins/complexes under investigation through the analyses of the elution volumes. The mixing of the proteins KCTD6^BTB^ and KCTD11^BTB^ with Cul3^NTD^ leaded to a profile characterized by two peaks. In addition to the peak corresponding to the excess of BTB domains (retention volume of ~ 14.5 mL), a second peak at ~ 10.5 mL appears (Fig 2A and 2B). This observation is indicative of the presence of a larger aggregate. The SDS-PAGE analysis of the peak content unveils the presence of both the BTB domains and Cul3^NTD^ (insets of Fig 2A and 2B). These findings clearly indicate the formation of stable complexes of these two KCTDs with Cul3. It is worth mentioning that the retention volume of these complexes falls between those observed, under identical experimental conditions, for aldolase (13,1 mL—Mw 160kDa) and for ferritin (9,1 mL—Mw 440kDa). Taking also into account the molecular weight of Cul3^NTD^ (42 kDa) and of KCTD6^BTB^ and KCTD11^BTB^ tetramers (~ 46 kDa), these findings are compatible with the formation of complexes with a 4:4 stoichiometry [(KCTD6^BTB^—Cul3^NTD^)_4_ and (KCTD11^BTB^—Cul3^NTD^)_4_]. On the other hand, the elution profiles of the mixtures formed by KCTD12^BTB^ and KCTD15^BTB^ with Cul3^NTD^ do not show the presence of high Mw species (Fig 2C and 2D). Indeed, in both cases a single peak at ~ 14.5 mL is observed. The SDS-PAGE analysis of this peak unveils the co-presence of either KCTD12^BTB^ and Cul3^NTD^ (insert of Fig 2C) or KCTD15^BTB^ and Cul3^NTD^ (insert of Fig 2D). On the basis of the retention volume of these peaks (~ 10.5 mL) and the Mw similarity of the tetrameric BTB domains (~ 46 kDa) and of Cul3^NTD^ (~ 42 kDa), it can be confidently assumed that the BTB domains of these two KCTD proteins do not form stable complexes with Cul3.

**Fig 3:**
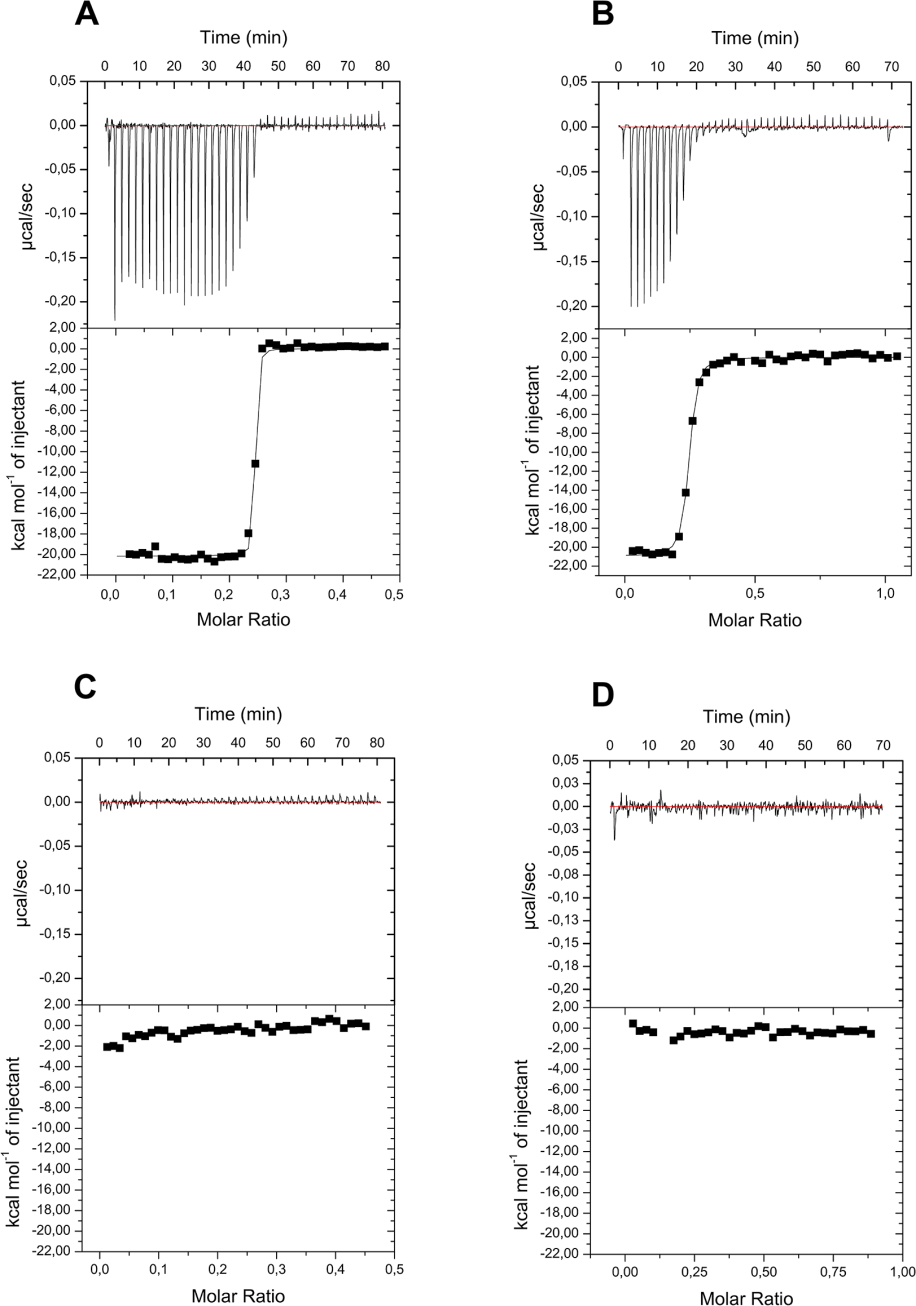
Quantification of Cul3-KCTDs binding by Isothermal Titration Calorimetry. ITC experiments were performed by titrating KCTD6^**BTB**^ (A), KCTD11^**BTB**^ (B), KCTD12^**BTB**^ (C) with Cul3^**NTD**^. For KCTD15^**BTB**^ the ITC experiment was reversed by titrating Cul3^**NTD**^ with KCTD15^**BTB**^ (D). The top and bottom panels report raw and integrated data, respectively.

In order to confirm and to quantify the data obtained from the gel filtration analyses, ITC experiments, which quantitative evaluate protein-protein binding through the analyses of the heat exchanges associated with their mixing. The heat exchanges upon titration of KCTD6^BTB^, KCTD11^BTB^, KCTD12^BTB^, and KCTD15^BTB^ with Cul3^NTD^ are shown in Fig 3. The negligible heat exchanges associated with the titration of KCTD12^BTB^ and KCTD15^BTB^ with this cullin suggest that no binding occurs (Fig 3C and 3D). This observation is in line with the gel filtration analyses. On the other hand, measurable heat exchanges are observed for the other two KCTD proteins (Fig 3A and 3B). The fitting of data with a single-binding site model demonstrates that both KCTD6^BTB^ and KCTD11^BTB^ form a tight complex with Cul3^NTD^. In particular the values of the dissociation constants K_D_ are 1,1 ± 0,6 nM (ΔH = -20.2 ± 0.1 kcal/mol) and 25 ± 2 nM (ΔH = -21.0 ± 0.1 kcal/mol) for KCTD6^BTB^/Cul3^NTD^ and KCTD11^BTB^/ Cul3^NTD^, respectively. In both cases, the value of the number of sites of KCTDs tetramers per Cul3^NTD^ is ~ 0.25. This analysis suggests that the two proteins form with Cul3^NTD^ the following complexes (KCTD6^BTB^–Cul3^NTD^)_4_ and (KCTD11^BTB^–Cul3^NTD^)_4_.

### Characterization of a KCTD11/KCTD12 chimera: role of the loop α2β3 in the Cul3 KCTDs interaction

**Fig 4:**
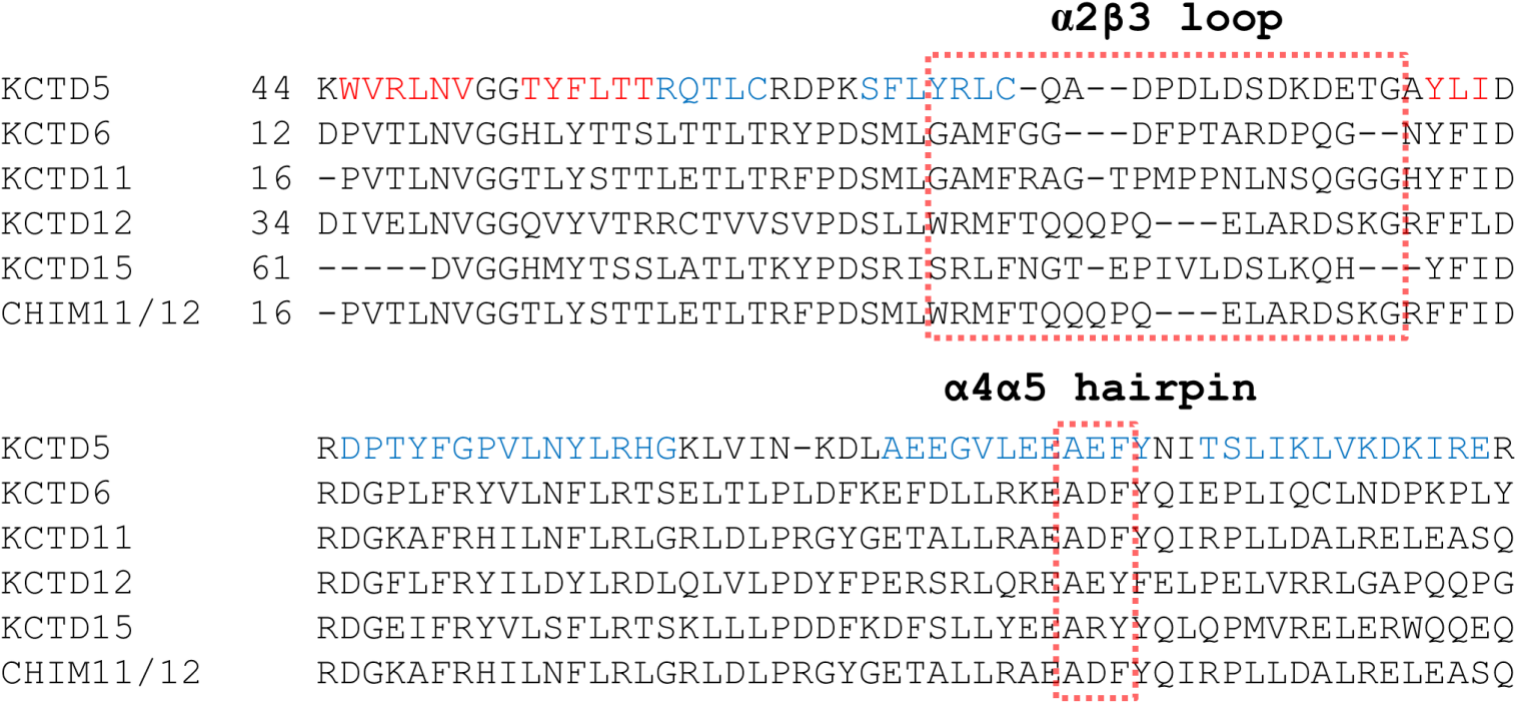
Multiple sequence alignment of the BTB domains of different KCTD proteins (KCTD5, KCTD6, KCTD11, KCTD12, KCTD15). The sequence of the novel chimeric construct CHIM11/12BTB is also reported.

**Fig 5:**
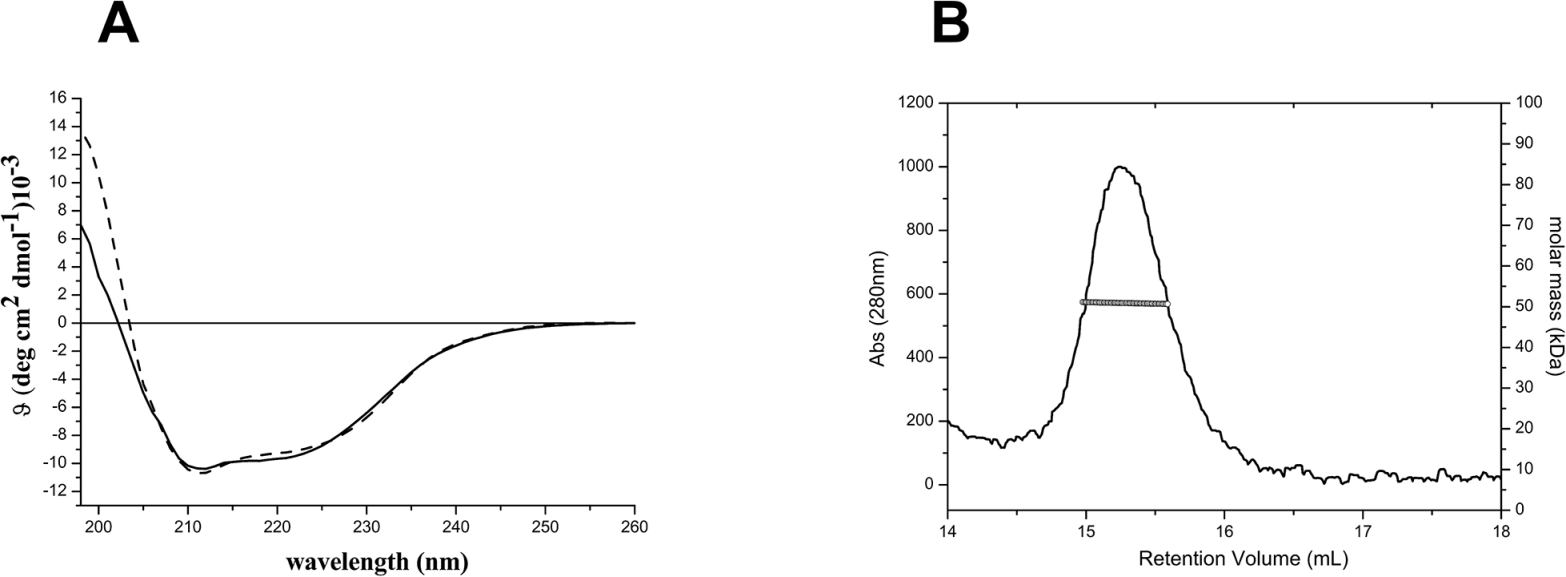
Biophysical characterization of CHIM11/12^BTB^ conducted by Far-UV CD spectroscopy (A) and by light scattering (B). The dashed line in (A) represents the far-UV CD spectrum of KCTD11^**BTB**^. The experiments were carried out in a 20mM sodium phosphate buffer (pH 7.5) containing 2 mM DTT.

**Fig 6:**
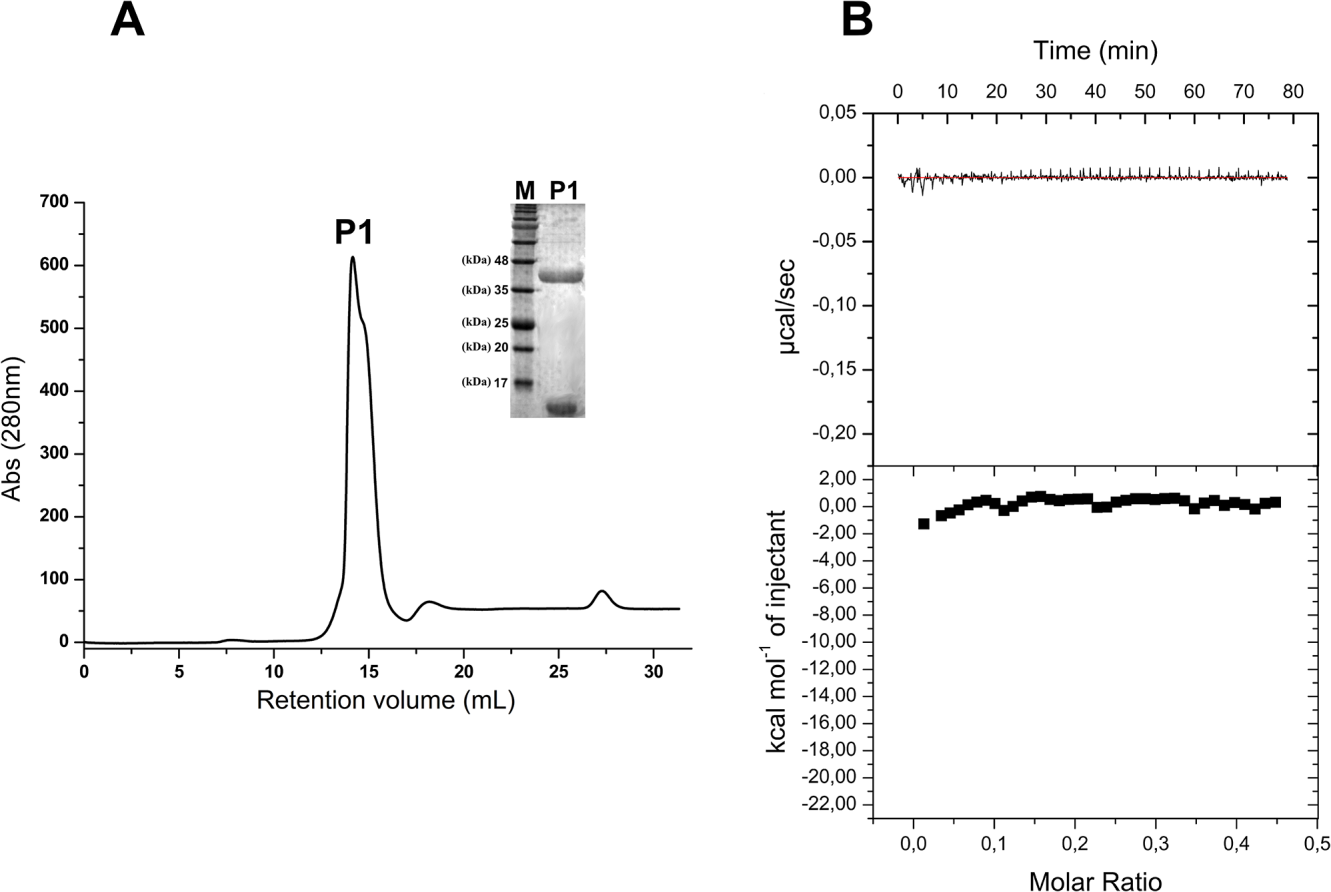
Detection and quantification of Cul3-CHIM11/12^BTB^ binding by gel filtration and ITC experiments. (A) Gel filtration elution profile of CHIM11/12^**BTB**^ after the mixing with Cul3^**NTD**^. (B) ITC characterization of the interaction of CHIM11/12^**BTB**^ with Cul3^**NTD**^. The insets report the SDS-PAGE analysis of the peaks.

Previous structural analyses have indicated that Cul3 recognition by KCTDs likely occurs through two main hot spots of the BTB domain: (a) the α2β3 loop and (b) the α4α5 helical hairpin [5, 6, 19]. In particular, residues of the α2β3 loop cooperate to Cul3 binding by establishing polar/electrostatic interactions whereas aromatic residues of the α4α5 helical hairpin pack against aromatic residues of Cul3 forming a tight Phe/Tyr cluster. A comparison of KCTD sequences shows that, with the exception of KCTD19, Tyr or Phe residues are present in the α4α5 fragment (S2 Fig). On the other hand, the sequence of the α2β3 loop is highly variable even within members of the same sub-group. The overall similarity of the α4α5 hairpin among KCTDs, independently of their ability to bind Cul3, indicates that the presence of aromatic residues in this region is not sufficient to warrant the cullin binding. Therefore, in order to gain further insights into the role played by specific KCTD regions in cullin recognition we focused the attention on the α2β3 loop. In this scenario, we designed and characterized a chimeric variant of KCTD11^BTB^/KCTD12^BTB^ (CHIM11/12^BTB^), in which the α2β3 loop of KCTD11 (residues 42–63) was replaced with the residues 61–80 of KCTD12, which are predicted to correspond to the α2β3 of this latter protein (Fig 4). This variant was expressed in *E*. *coli* and purified as a pure and homogenous product. The far-UV CD spectrum of CHIM11/12^BTB^ is virtually identical to that observed for KCTD11^BTB^ (Fig 5A). Moreover, light scattering experiments show that also the tetrameric association is preserved in the chimeric form. Indeed, the experimental value of the molecular weight of CHIM11/12^BTB^ (62.4 ± 0.5 kDa) is in close to the value expected for the tetramer (62 kDa) (Fig 5B). These findings suggest that the insertion of this KCTD12 loop in the KCTD11 scaffold does not perturb the overall structure of the protein and its oligomeric state. Gel filtration analyses revealed that CHIM11/12^BTB^, differently from KCTD11^BTB^, is not able to interact with Cul3 (Fig 6A). The lack of binding between CHIM11/12^BTB^ and Cul3 is in line with the data emerged from the ITC analysis (Fig 6B).

### Molecular dynamics simulations of KCTD11^BTB^ and CHIM11/12^BTB^

**Fig 7:**
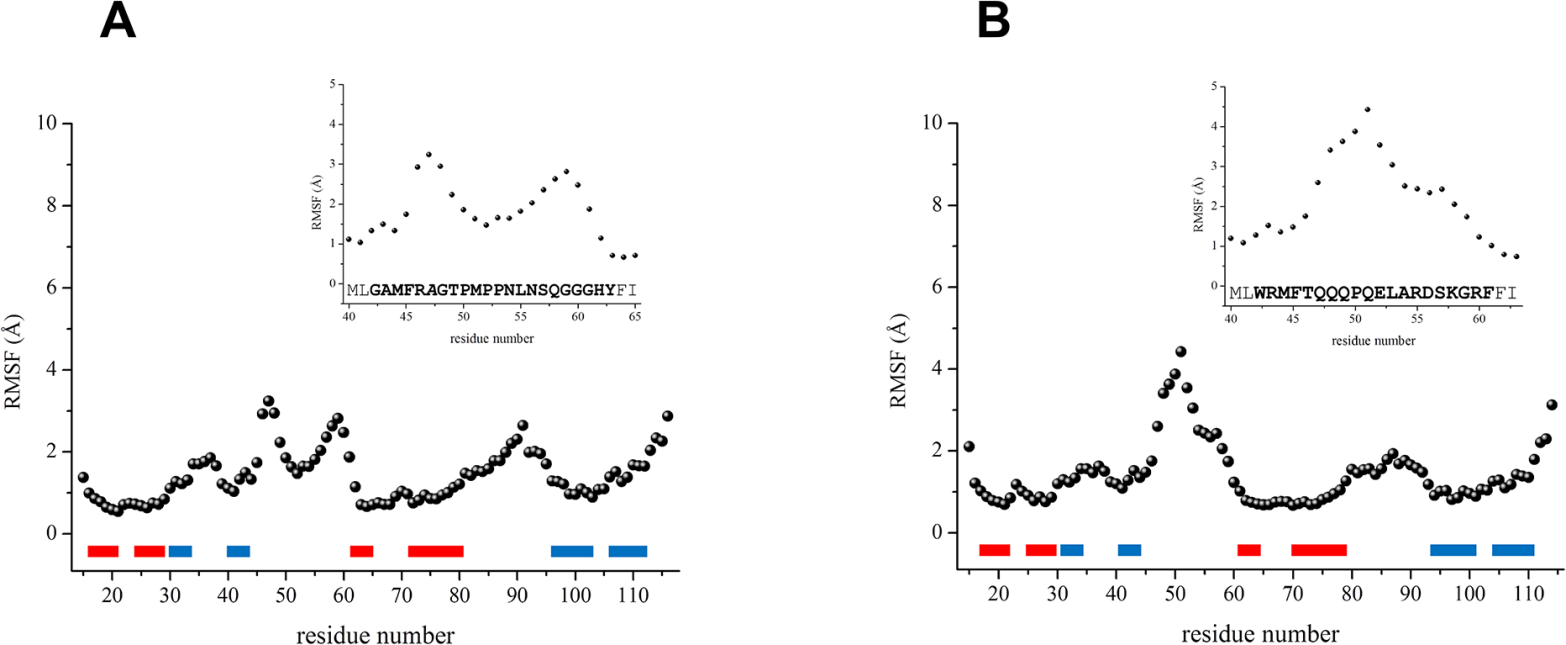
Root mean square fluctuations per residue of KCTD11^BTB^ and CHIM11/12^BTB^. RMSF values calculated on C^α^ atoms in the equilibrated region the trajectories (20–100 ns) for the simulations carried out on KCTD11^**BTB**^ (A) and CHIM11/12^**BTB**^ (B). Secondary structure elements are represented as bars. Helices and strands are colored in blue and red, respectively. In the insets the RMSF values of the α2β3 loops, within the different amino acid sequences, are reported.

In order to gain structural insights into the radically different behavior of KCTD11^BTB^ and CHIM11/12^BTB^ in Cul3 recognition, we performed molecular dynamics simulations on these two proteins focusing the attention on the dynamic properties of the α2β3 loop. The starting models used in the simulations were generated as described in the Materials and Methods section. The analysis of the indicators commonly adopted to check system stability in MD analyses indicates that both structures are stable in the simulation timescale (100 ns). This is clearly indicated by the time evolution of (a) the root mean square deviations (RMSD) of the trajectory structures from the starting model, (b) the gyration radius, (c) the secondary structure, and (d) the total number of hydrogen bonds (S3–S6 Figs). It is worth mentioning that the helix α2 is endowed with a rather dynamical behavior in both KCTD11^BTB^ and CHIM11/12^BTB^. The convergence of the simulations was also assessed by computing the RMSIP (see the methods for the definition) between two halves of the equilibrated trajectory (20–60 and 60–100 ns). The values of the RMSIP, which are 0.70 and 0.71 for KCTD11^BTB^ and CHIM11/12^BTB^, respectively, indicate that both simulations reached a satisfactory level of convergence. The observed stability of CHIM11/12^BTB^, in line with the biophysical characterization of this variant, indicates that the KCTD12^BTB^ α2β3 loop can be effortlessly accommodated in KCTD11^BTB^ tetramer. It is important to note that both KCTD11^BTB^ and CHIM11/12^BTB^ tetramers are stabilized by electrostatic and H-bonds interactions. Among those, particularly relevant is the role of Asp66 and Arg80 (Asp64 and Arg78 of CHIM11/12^BTB^ sequence), whose side chains are involved in conserved electrostatic interactions (S7 Fig).

a)Insights into the local mobility of different protein regions have been obtained by the analysis of the root mean square fluctuation (RMSF) values calculated on C^α^ atoms in the equilibrated region the trajectories (20–100 ns) (Fig 7). As a general trend, residues belonging to structural elements of the proteins exhibit a lower flexibility with RMSF values of ~ 1 Å or below; on the other hand loop and terminal residues display much higher RMSF values (up to 3–4 Å). Notably, in both simulations considerable fluctuations are exhibited by residues of the α2β3 loop. However a closer inspection of the RMSF values indicates that the flexibility of the two α2β3 loops is clearly context-dependent. Indeed, the α2β3 loop of CHIM11/12^BTB^ exhibits a higher flexibility. The somewhat reduced mobility of this loop in KCTD11^BTB^ may be ascribed to the presence of a proline-rich fragment (residues 50–53 PMPP) in the α2β3 central region of the protein sequence.

**Fig 8:**
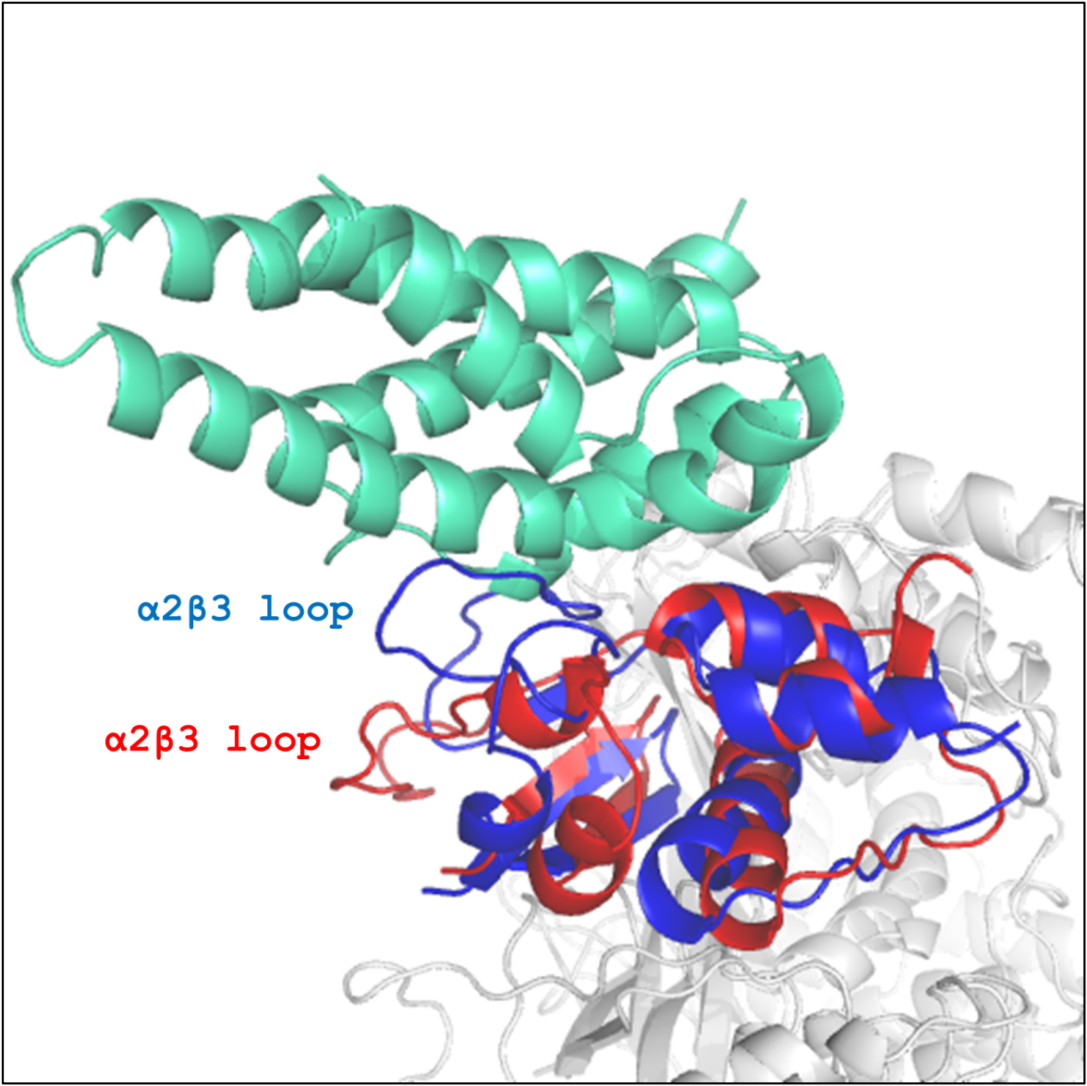
Cul3 binding site in the average MD structures of KCTD11^BTB^ and CHIM11/12^BTB^. KCTD11^**BTB**^, CHIM11/12^**BTB**^, Cul3^**NTD**^ are represented in blue, red and green, respectively. For clarity, a single chain of the tetramers is highlighted.

The differential dynamic behavior of the α2β3 loop in KCTD11^BTB^ and CHIM11/12^BTB^ is also confirmed by an essential dynamics analysis carried out by using the procedure developed by Amadei et al. (see also the Methods section). The analysis of the collective motions of the proteins shows that most of the atomic displacements are contained in the first few eigenvectors. Indeed, the essential subspace spanned by the first 10 eigenvectors covers about 73% and 75% of the total fluctuations in KCTD11^BTB^ and CHIM11/12^BTB^ dynamics, respectively. A film-like fashion representation of the motion along the first eigenvector, which represents as much as 37% and 43% of the total KCTD11^BTB^ and CHIM11/12^BTB^ fluctuations, respectively, is reported in S8 Fig. A distinctive feature of the dynamic behavior of the two α2β3 loops is the presence of a rather rigid central region in KCTD11^BTB^ (S8 Fig). These observations open the possibility that the high flexibility of this loop in CHIM11/12^BTB^ may lead to the obstruction of the cullin binding site, thus preventing the formation of the complex. However, the analysis of trajectory structures indicates that this is not the case. Indeed, the analysis of the binding site in the average MD structures of KCTD11^BTB^ and CHIM11/12^BTB^ indicates that the site is available for binding in both cases (Fig 8). This suggests that the interactions established by the α2β3 loop of KCTDs are essential for Cul3 recognition. The elevated flexibility of this loop in CHIM11/12^BTB^ can make the formation of stable interaction more difficult, thus contributing to the lack of binding of the protein to Cul3.

## Discussion

The KCTD family is an emerging class of BTB-containing proteins involved in important biological processes. Very recent studies have highlighted that these proteins, although sharing significant molecular features, play diversified roles. Since the BTB domain is an important module implicated in Cul3 recognition [31,32], we here evaluated and/or quantified the binding of this protein to the BTB of KCTD proteins, which are known to be involved either in cullin-mediated (KCTD6 and KCTD11) or in cullin-independent (KCTD12 and KCTD15) activities.

The quantification of the binding of Cul3 to KCTD6^BTB^ and KCTD11^BTB^ indicates that these two proteins are endowed with a high affinity for this cullin. Indeed, KCTD6^BTB^ tightly binds Cul3 with K_D_ of 1,1 nM; KCTD11^BTB^ exhibits a slightly lower affinity for the cullin (K_D_ of 25 nM). Interestingly, both proteins recognize Cul3 forming stable complexes, which can be easily purified by gel filtration chromatography, with a 4:4 stoichiometry. The affinity of the BTB domains of these tetrameric KCTDs for Cul3 is comparable to that reported for the pentameric KCTD5^BTB^ [19] and much higher than those reported for other BTB domains endowed with a lower oligomeric organization [25,33]. Since a reliable model of the complex of KCTD11^BTB^ with Cul3 has suggested that the cullin binds at the intersubunit interface of the protein tetramer [5,6], it is likely that also KCTD6^BTB^ recognizes this cullin at the tetramer interface. Collectively, these observations suggest that the inter-subunit interface of both KCTD tetramers and pentamers provide large surfaces for tight Cul3 binding. Current data suggest that in monomeric or dimeric BTB-containing proteins a similar tight Cul3 binding is achieved with the contribution of other structural motifs such as the 3-box [25].

ITC and Gel filtration analyses indicate that both KCTD12^BTB^ and KCTD15^BTB^ are unable to bind Cul3. Although our data were derived using recombinant proteins expressed in a bacterial system, to the best of our knowledge (see also Ref [2]), these results represent the first experimental proof that some members of the KCTD family do not recognize Cul3. This finding is somewhat surprising considering (a) that these proteins present elevated sequence identities with the BTB domains of Cul3-interacting KCTDs (range from 30 to 57%) (see Table 1 for details) and (b) that KCTD12 was reported to be a potential Cul3 interactor in a recent proteomic study [18]. These observations clearly indicate that the involvement of KCTD12 and KCTD15 in cullin-independent activities have led the loss of their ability to bind Cul3. Taking into account the close similarity of KCTD8^BTB^ and KCTD16^BTB^ with KCTD12^BTB^ and of KCTD1^BTB^ with KCTD15^BTB^ it is likely that also these closely related proteins are unable to recognize Cul3. Intriguingly, a deeper inspection of the sequence comparison (Table 1 and S2 Fig) and of the KCTD gene family evolutionary tree reported by Baranova and coworkers (Fig 1A of reference [2]) unravels that the sequence of the non-Cul3 binding KCTD15^BTB^ is closer to the Cul3 binding KCTD6^BTB^ (57% sequence identity) than to KCTD12^BTB^ (42% sequence identity) that is also unable to recognize the cullin. In other words, the sequences of cullin binding and cullin independent KCTDs are not segregated in the evolutionary tree. These considerations indicate that the capability of KCTD proteins to recognize Cul3 has been lost more than once in distinct events along the evolution. In particular, the loss of ability in Cul3 recognition may have occurred in the divergence of KCTD12 from KCTD6 and KCTD15 and then in the separation of KCTD15 and KCTD6.

**Table 1:** Percentages of sequence identity and number of aligned residues of the BTB domains of selected KCTD members are reported on the right and left side of the diagonal, respectively.

BTB domain	KCTD5	KCTD6	KCTD7	KCTD11	KCTD12	KCTD13	KCTD15	BTBD10	SHKBP1
**KCTD5**		41%	35%	40%	34%	36%	34%	24%	41%
**KCTD6**	96		47%	61%	44%	43%	57%	28%	45%
**KCTD7**	95	98		43%	34%	47%	39%	28%	41%
**KCTD11**	100	99	100		40%	43%	43%	29%	42%
**KCTD12**	92	100	99	103		36%	42%	27%	34%
**KCTD13**	94	90	92	94	91		30%	33%	34%
**KCTD15**	91	90	94	100	97	97		24%	43%
**BTBD10**	67	90	69	90	73	52	59		28%
**SHKBP1**	103	92	93	97	96	104	91	68	

In addition to the proteins here characterized (KCTD6, KCTD11, KCTD12, and KCTD15), we included into the comparison representative members (KCTD5, KCTD7, KCTD13, BTBD10 and SHKBP1) of KCTD subgroups whose interaction with Cul3 has been experimentally demonstrated.

Present findings also provide insights into this intricate KCTDs-Cul3 recognition process. Although experimental structural characterizations of these complexes are not available, modeling and molecular dynamics studies have suggested that both pentameric and tetrameric KCTDs recognize Cul3 though the α2β3 loop and the α4α5 helical hairpin regions [5,19]. The large conservation of the aromatic residues in the α4α5 helical hairpin among both cullin binding and cullin-independent KCTDs and the inability of the chimeric protein CHIM11/12^BTB^ to bind Cul3 indicate that the α2β3 loop is crucial for cullin recognition. It is worth mentioning that present MD studies indicate that the inability of CHIM11/12^BTB^ to bind Cul3 cannot be ascribed to the obstruction of the binding site by the α2β3 KCTD12 sequence. Therefore, in KCTD11 and other Cul3-binding KCTDs the α2β3 loop contributes to Cul3 recognition by forming interactions that directly stabilize the complex. The high variability of this region (S2 Fig) among cullin binding KCTDs suggests that different sequences of the loop may contribute to the binding. The high variability of the loop has also been used in the evolution to switch off/on the ability to bind Cul3.

Present findings represent a significant advancement in the recognition process of Cul3 by KCTDs. Taking into account the close similarity of KCTD proteins belonging to the same clade of the evolutionary tree (Fig 1A of reference [2]), our results indicate that members of the Clade A (KCTD15 and KCTD1) and F (KCTD12, KCTD8 and KCTD16) do not interact with Cul3. We also show that members of the Clade B (KCTD6, KCTD11, and KCTD21) strongly bind to Cul3 as previously indicated for the members of the Clade E (KCTD2, KCTD5, and KCTD17) [14,19]. Taking into account the extensive literature functional and biochemical characterization of the Cul3 interaction with members of the clade C (KCTD10, KCTD13 and TNFAIP1) [8], of the clade G (BTBD10 and KCTD20) [18,34], and of the clade D (KCTD3 and SHKBP1) [19,34], present findings provide a virtually complete coverage of the characterization of Cul3 binding ability of the entire KCTD family.

## Supporting Information

S1 Fig Far-UV CD spectra of KCTD6^BTB^ (A), KCTD11^BTB^ (B), and KCTD12^BTB^ (C). (TIFF)

S2 Fig Alignment of sequences of the members of KCTD family. The sequence number of the first residue of the BTB domain of each protein is reported. KCTD19a and KCTD19b refer to the two BTB domains of this protein. Helices and strands of KCTD5^BTB^ are highlighted in blue and red, respectively. The hotspots for cullin recognition are also highlighted.
(TIF)

S3 Fig Cα RMSD values of trajectory structures from the starting models of KCTD11^BTB^ (A) and CHIM11/12^BTB^ (B). (TIF)

S4 Fig Time evolution of the gyration radius of KCTD11^BTB^ (black) and CHIM11/12^BTB^ (red) in the MD simulations. (TIF)

S5 Fig Time evolution of the secondary structure elements of KCTD11^BTB^ (A) and CHIM11/12^BTB^ (B) in the MD simulation. (TIF)

S6 Fig Time evolution of the total number of H-bond interactions in trajectory structures of KCTD11^BTB^ (black) and CHIM11/12^BTB^ (red). (TIF)

S7 Fig Evolution of the distances between pairs of atoms that form H-bond (Gly22^O^-Ser27^N^ in KCTD11^BTB^ (A) and CHIM11/12^BTB^ (B)) or electrostatic (Asp66^Oδ1^-Arg80^Nη2^ in KCTD11^BTB^ (C), Asp64^Oδ1^-Arg78^Nη2^ in CHIM11/12^BTB^ (D) Asp68^Oδ1^-Arg73^Nη1^ in KCTD11^BTB^ (E), Asp66^Oδ2^-Arg71^Nη1^ in CHIM11/12^BTB^ (F)) interactions involved in the stabilization of the tetramers. (TIF)

S8 Fig C^α^ trace motions of KCTD11^BTB^ (A) and CHIM11/12^BTB^ (B) as derived from the essential dynamics analysis. The differentiated motions along the first eigenvector are represented in a film-like fashion. Large movements are displayed by the α2-β3 loop region. An arbitrary color scale (from violet to red) is used to represent the movement. For clarity, the motion of a single chain within the tetramers is shown.
(TIF)

## References

[1] Stogios PJ, Downs GS, Jauhal JJ, Nandra SK, Prive GG (2005) Sequence and structural analysis of BTB domain proteins. Genome biology 6: R82 16207353 10.1186/gb-2005-6-10-r82 PMC1257465

[2] Skoblov M, Marakhonov A, Marakasova E, Guskova A, Chandhoke V, Birerdinc A, et al (2013) Protein partners of KCTD proteins provide insights about their functional roles in cell differentiation and vertebrate development. BioEssays: news and reviews in molecular, cellular and developmental biology 35: 586–596. 10.1002/bies.201300002 23592240

[3] Liu Z, Xiang Y, Sun G (2013) The KCTD family of proteins: structure, function, disease relevance. Cell & bioscience 3: 45. 24268103 10.1186/2045-3701-3-45 PMC3882106

[4] Birerdinc A, Nohelty E, Marakhonov A, Manyam G, Panov I, Coon S, et al (2010) Pro-apoptotic and antiproliferative activity of human KCNRG, a putative tumor suppressor in 13q14 region. Tumour biology: the journal of the International Society for Oncodevelopmental Biology and Medicine 31: 33–45. 10.1007/s13277-009-0005-0 20237900 PMC2803748

[5] Correale S, Pirone L, Di Marcotullio L, De Smaele E, Greco A, Mazzà D, et al (2011) Molecular organization of the cullin E3 ligase adaptor KCTD11. Biochimie 93: 715–724. 10.1016/j.biochi.2010.12.014 21237243

[6] Canettieri G, Di Marcotullio L, Greco A, Coni S, Antonucci L, Infante P, et al (2010) Histone deacetylase and Cullin3-REN(KCTD11) ubiquitin ligase interplay regulates Hedgehog signalling through Gli acetylation. Nature cell biology 12: 132–142. 10.1038/ncb2013 20081843

[7] De Smaele E, Di Marcotullio L, Moretti M, Pelloni M, Occhione MA, Infante P, et al (2011) Identification and characterization of KCASH2 and KCASH3, 2 novel Cullin3 adaptors suppressing histone deacetylase and Hedgehog activity in medulloblastoma. Neoplasia 13: 374–385. 21472142 10.1593/neo.101630 PMC3071086

[8] Chen Y, Yang Z, Meng M, Zhao Y, Dong N, Yan H, et al (2009) Cullin mediates degradation of RhoA through evolutionarily conserved BTB adaptors to control actin cytoskeleton structure and cell movement. Molecular cell 35: 841–855. 10.1016/j.molcel.2009.09.004 19782033

[9] Ding X, Luo C, Zhou J, Zhong Y, Hu X, Zhou F, et al (2009) The interaction of KCTD1 with transcription factor AP-2alpha inhibits its transactivation. Journal of cellular biochemistry 106: 285–295. 10.1002/jcb.22002 19115315

[10] Golzio C, Willer J, Talkowski ME, Oh EC, Taniguchi Y, Jacquemont S, et al (2012) KCTD13 is a major driver of mirrored neuroanatomical phenotypes of the 16p11.2 copy number variant. Nature 485: 363–367. 10.1038/nature11091 22596160 PMC3366115

[11] Pfeiffenberger C, Allada R (2012) Cul3 and the BTB adaptor insomniac are key regulators of sleep homeostasis and a dopamine arousal pathway in Drosophila. PLoS genetics 8: e1003003 10.1371/journal.pgen.1003003 23055946 PMC3464197

[12] Schwenk J, Metz M, Zolles G, Turecek R, Fritzius T, Bildl W, et al (2010) Native GABA(B) receptors are heteromultimers with a family of auxiliary subunits. Nature 465: 231–235. 10.1038/nature08964 20400944

[13] Bartoi T, Rigbolt KT, Du D, Kohr G, Blagoev B, Kornau HC (2010) GABAB receptor constituents revealed by tandem affinity purification from transgenic mice. The Journal of biological chemistry 285: 20625–20633. 10.1074/jbc.M109.049700 20406808 PMC2898358

[14] Bayon Y, Trinidad AG, de la Puerta ML, Del Carmen Rodriguez M, Bogetz J, Rojas A, et al (2008) KCTD5, a putative substrate adaptor for cullin3 ubiquitin ligases. The FEBS journal 275: 3900–3910. 10.1111/j.1742-4658.2008.06537.x 18573101

[15] Zarelli VE, Dawid IB (2013) Inhibition of neural crest formation by Kctd15 involves regulation of transcription factor AP-2. Proceedings of the National Academy of Sciences of the United States of America 110: 2870–2875. 10.1073/pnas.1300203110 23382213 PMC3581937

[16] Turecek R, Schwenk J, Fritzius T, Ivankova K, Zolles G, Adelfinger L, et al (2014) Auxiliary GABAB receptor subunits uncouple G protein betagamma subunits from effector channels to induce desensitization. Neuron 82: 1032–1044. 10.1016/j.neuron.2014.04.015 24836506

[17] Adelfinger L, Turecek R, Ivankova K, Jensen AA, Moss SJ, Gassmann M, et al (2014) GABAB receptor phosphorylation regulates KCTD12-induced K(+) current desensitization. Biochemical pharmacology 91: 369–379. 10.1016/j.bcp.2014.07.013 25065880 PMC4402209

[18] Bennett EJ, Rush J, Gygi SP, Harper JW (2010) Dynamics of cullin-RING ubiquitin ligase network revealed by systematic quantitative proteomics. Cell 143: 951–965. 10.1016/j.cell.2010.11.017 21145461 PMC3008586

[19] Balasco N, Pirone L, Smaldone G, Di Gaetano S, Esposito L, Pedone EM, et al (2014) Molecular recognition of Cullin3 by KCTDs: insights from experimental and computational investigations. Biochimica et biophysica acta 1844: 1289–1298. 10.1016/j.bbapap.2014.04.006 24747150

[20] Di Marcotullio L, Ferretti E, De Smaele E, Argenti B, Mincione C, Zazzeroni F, et al (2004) REN(KCTD11) is a suppressor of Hedgehog signaling and is deleted in human medulloblastoma. Proceedings of the National Academy of Sciences of the United States of America 101: 10833–10838. 15249678 10.1073/pnas.0400690101 PMC490020

[21] Gallo R, Zazzeroni F, Alesse E, Mincione C, Borello U, Buanne P, et al (2002) REN: a novel, developmentally regulated gene that promotes neural cell differentiation. The Journal of cell biology 158: 731–740. 12186855 10.1083/jcb.200202024 PMC2174014

[22] Lange S, Perera S, Teh P, Chen J (2012) Obscurin and KCTD6 regulate cullin-dependent small ankyrin-1 (sAnk1.5) protein turnover. Molecular biology of the cell 23: 2490–2504. 10.1091/mbc.E12-01-0052 22573887 PMC3386213

[23] Pirone L, Esposito C, Correale S, Graziano G, Di Gaetano S, Vitagliano L, et al (2013) Thermal and chemical stability of two homologous POZ/BTB domains of KCTD proteins characterized by a different oligomeric organization. BioMed research international 2013: 162674 10.1155/2013/162674 24307990 PMC3838848

[24] Correale S, Esposito C, Pirone L, Vitagliano L, Di Gaetano S, Pedone E (2013) A biophysical characterization of the folded domains of KCTD12: insights into interaction with the GABAB2 receptor. Journal of molecular recognition: JMR 26: 488–495. 10.1002/jmr.2291 23996491

[25] Errington WJ, Khan MQ, Bueler SA, Rubinstein JL, Chakrabartty A, Privé GG (2012) Adaptor protein self-assembly drives the control of a cullin-RING ubiquitin ligase. Structure 20: 1141–1153. 10.1016/j.str.2012.04.009 22632832

[26] de Paola I, Pirone L, Palmieri M, Balasco N, Esposito L, Russo L, et al (2015) Cullin3—BTB interface: a novel target for stapled peptides. PloS one 10: e0121149 10.1371/journal.pone.0121149 25848797 PMC4388676

[27] Van Der Spoel D, Lindahl E, Hess B, Groenhof G, Mark AE, Berendsen HJ (2005) GROMACS: fast, flexible, and free. Journal of computational chemistry 26: 1701–1718. 16211538 10.1002/jcc.20291

[28] Amadei A, Linssen AB, Berendsen HJ (1993) Essential dynamics of proteins. Proteins 17: 412–425. 8108382 10.1002/prot.340170408

[29] Merlino A, Vitagliano L, Ceruso MA, Di Nola A, Mazzarella L (2002) Global and local motions in ribonuclease A: a molecular dynamics study. Biopolymers 65: 274–283. 12382288 10.1002/bip.10225

[30] Humphrey W, Dalke A, Schulten K (1996) VMD: visual molecular dynamics. Journal of molecular graphics 14: 33–38, 27–38. 8744570 10.1016/0263-7855(96)00018-5

[31] Xu L, Wei Y, Reboul J, Vaglio P, Shin TH, Vidal M, et al (2003) BTB proteins are substrate-specific adaptors in an SCF-like modular ubiquitin ligase containing CUL-3. Nature 425: 316–321. 13679922 10.1038/nature01985

[32] Pintard L, Willems A, Peter M (2004) Cullin-based ubiquitin ligases: Cul3-BTB complexes join the family. The EMBO journal 23: 1681–1687. 15071497 10.1038/sj.emboj.7600186 PMC394240

[33] Canning P, Cooper CD, Krojer T, Murray JW, Pike AC, Chaikuad A, et al (2013) Structural basis for Cul3 protein assembly with the BTB-Kelch family of E3 ubiquitin ligases. The Journal of biological chemistry 288: 7803–7814. 10.1074/jbc.M112.437996 23349464 PMC3597819

[34] Wang J, Huo K, Ma L, Tang L, Li D, Huang X, et al (2011) Toward an understanding of the protein interaction network of the human liver. Molecular systems biology 7: 536 10.1038/msb.2011.67 21988832 PMC3261708

