# Supplementary figures and images for: Cullin 3 Recognition Is Not a Universal Property among KCTD Proteins
Source: PLoS One. 2015 May 14;10(5):e0126808. doi: 10.1371/journal.pone.0126808 (PMC4431850; doi:10.1371/journal.pone.0126808)

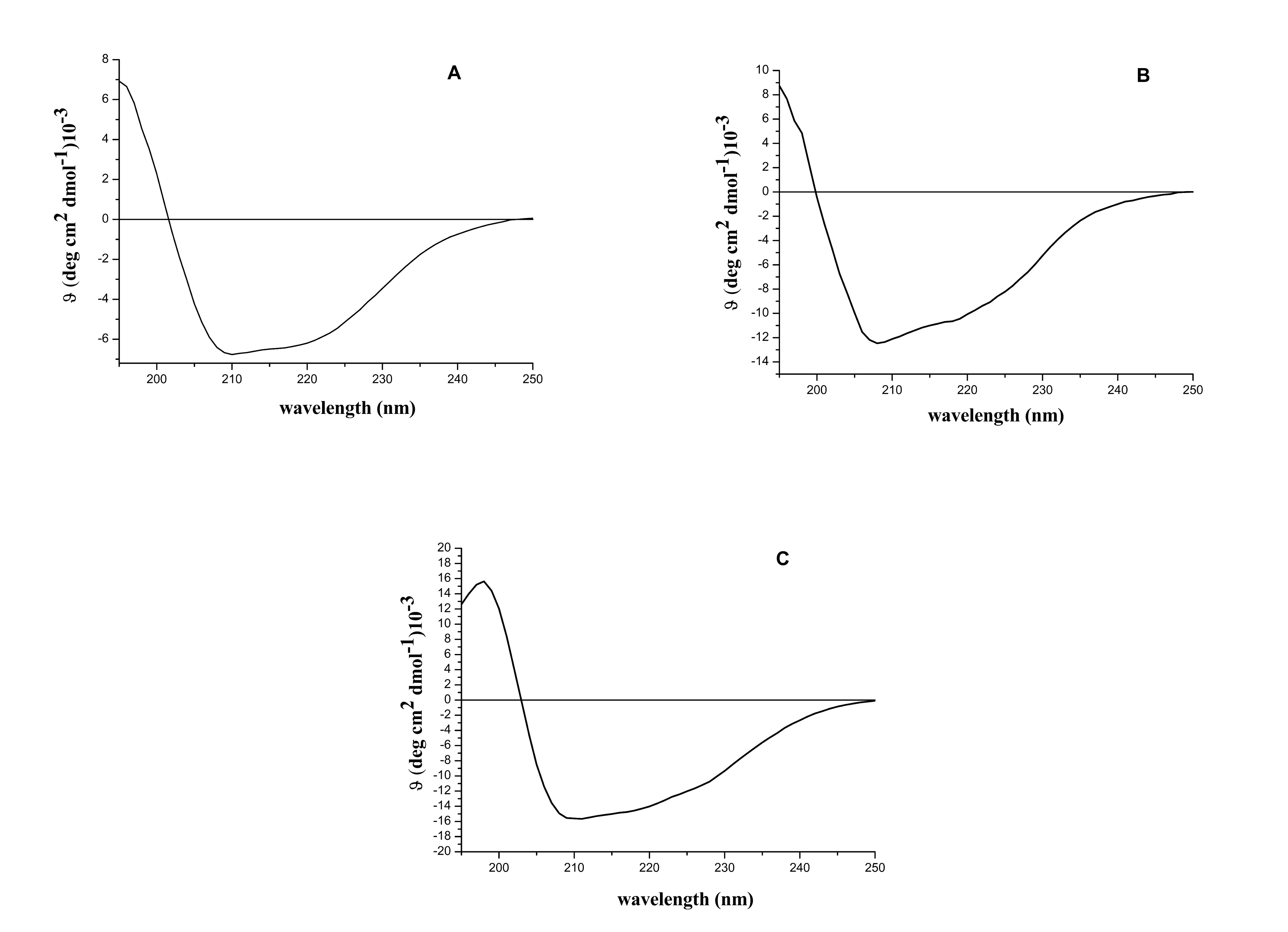

Supplement: S1 Fig — (TIFF) [file pone.0126808.s001.tiff]

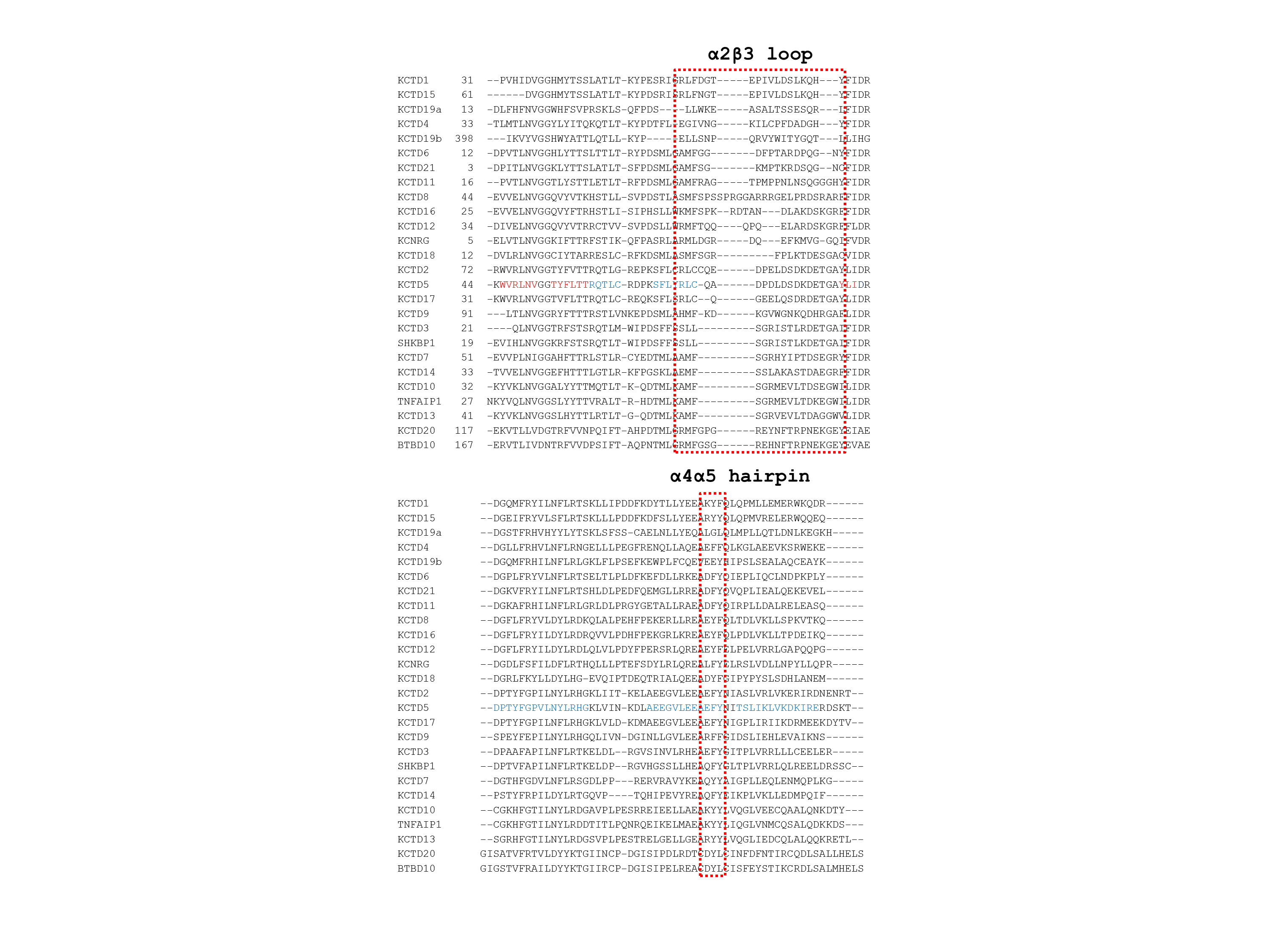

Supplement: S2 Fig — The sequence number of the first residue of the BTB domain of each protein is reported. KCTD19a and KCTD19b refer to the two BTB domains of this protein. Helices and strands of KCTD5BTB are highlighted in blue and red, respectively. The hotspots for cullin recognition are also highlighted. (TIF) [file pone.0126808.s002.tif]

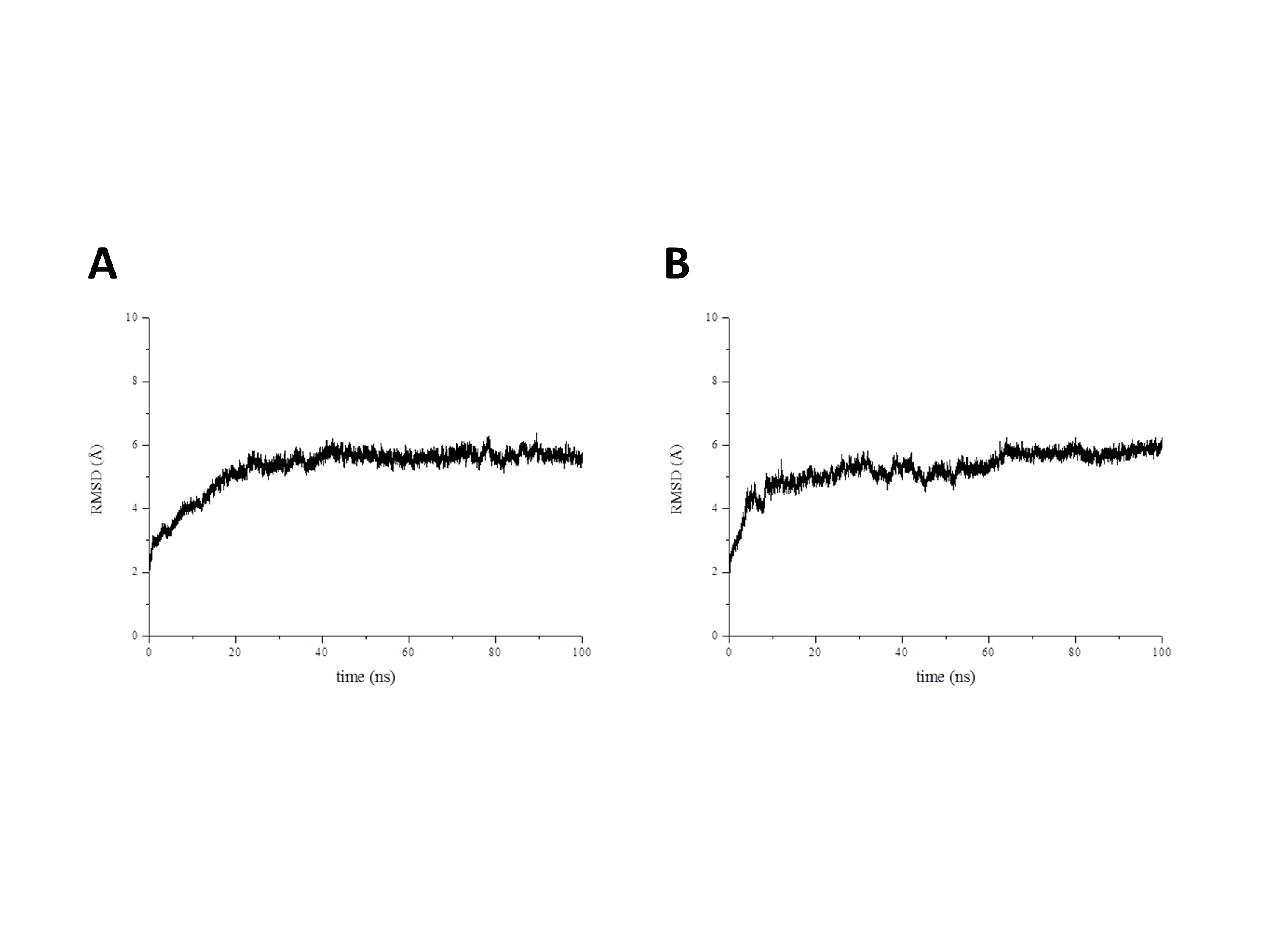

Supplement: S3 Fig — (TIF) [file pone.0126808.s003.tif]

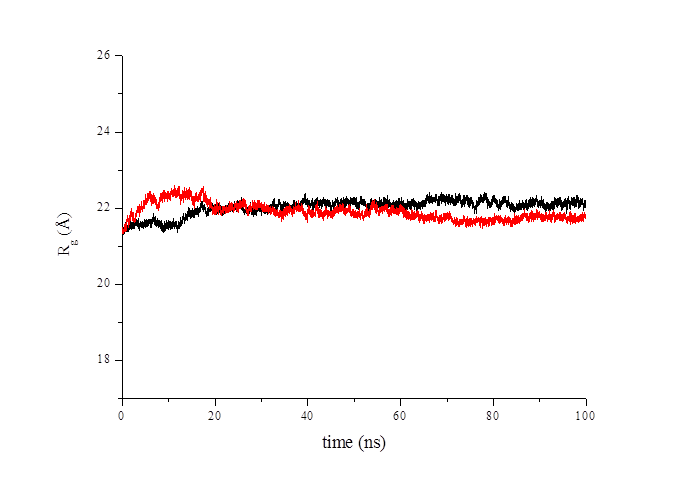

Supplement: S4 Fig — (TIF) [file pone.0126808.s004.tif]

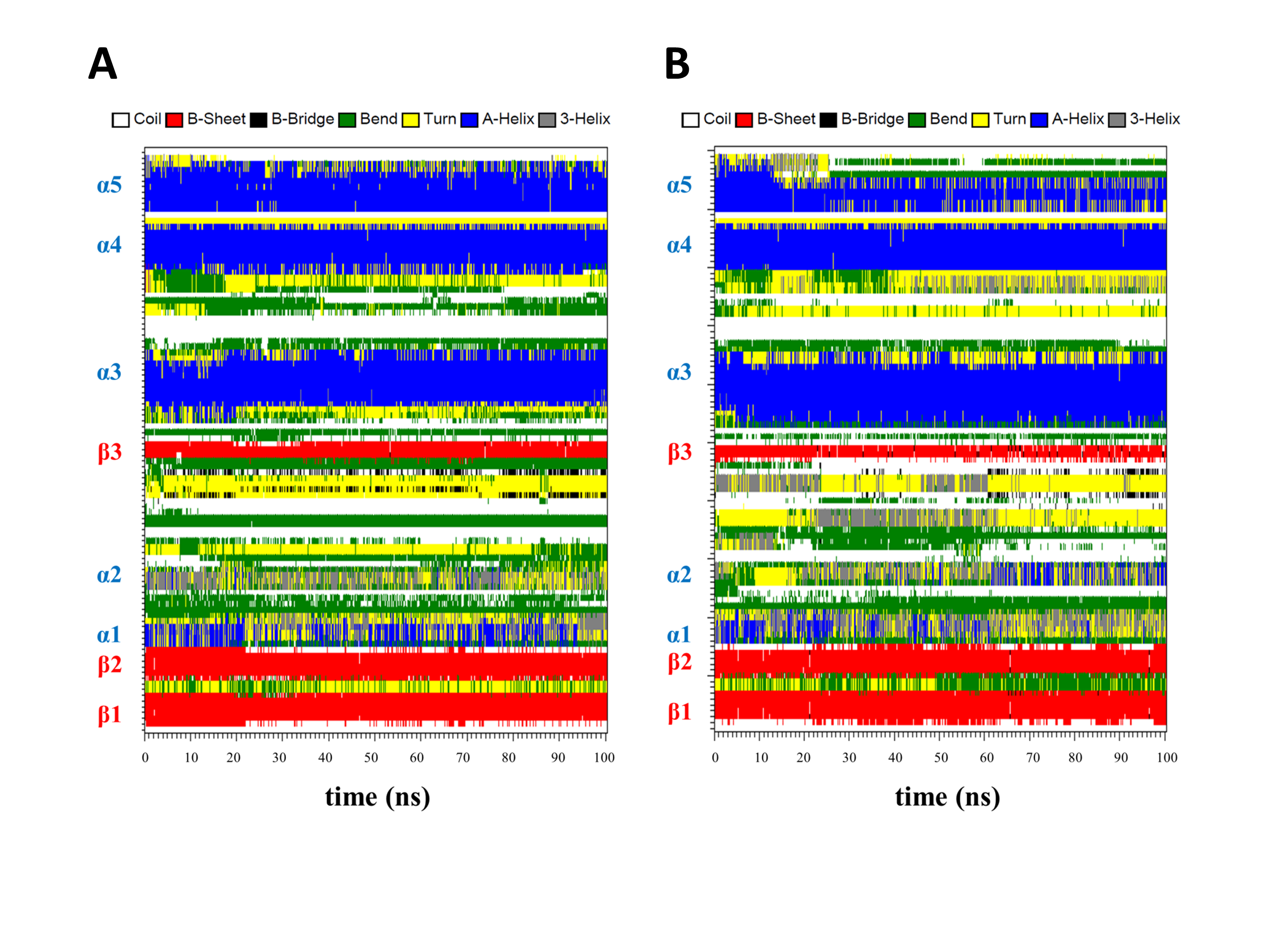

Supplement: S5 Fig — (TIF) [file pone.0126808.s005.tif]

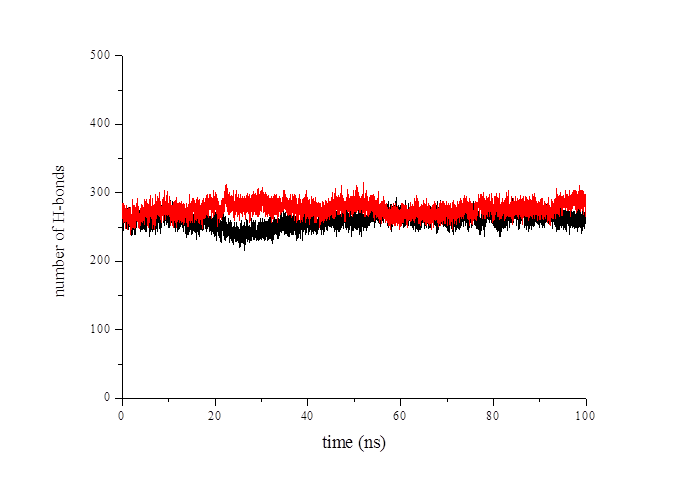

Supplement: S6 Fig — (TIF) [file pone.0126808.s006.tif]

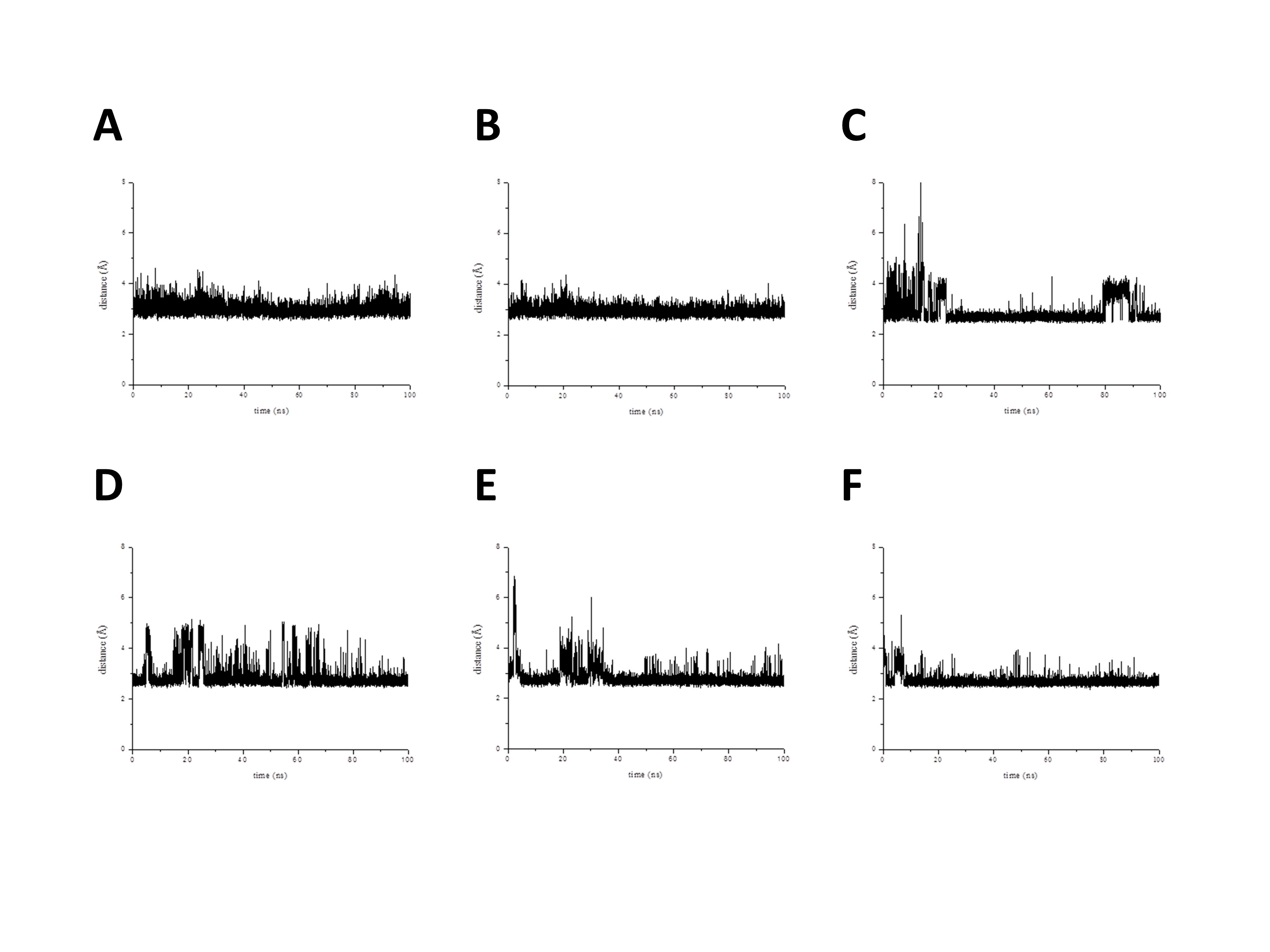

Supplement: S7 Fig — (TIF) [file pone.0126808.s007.tif]

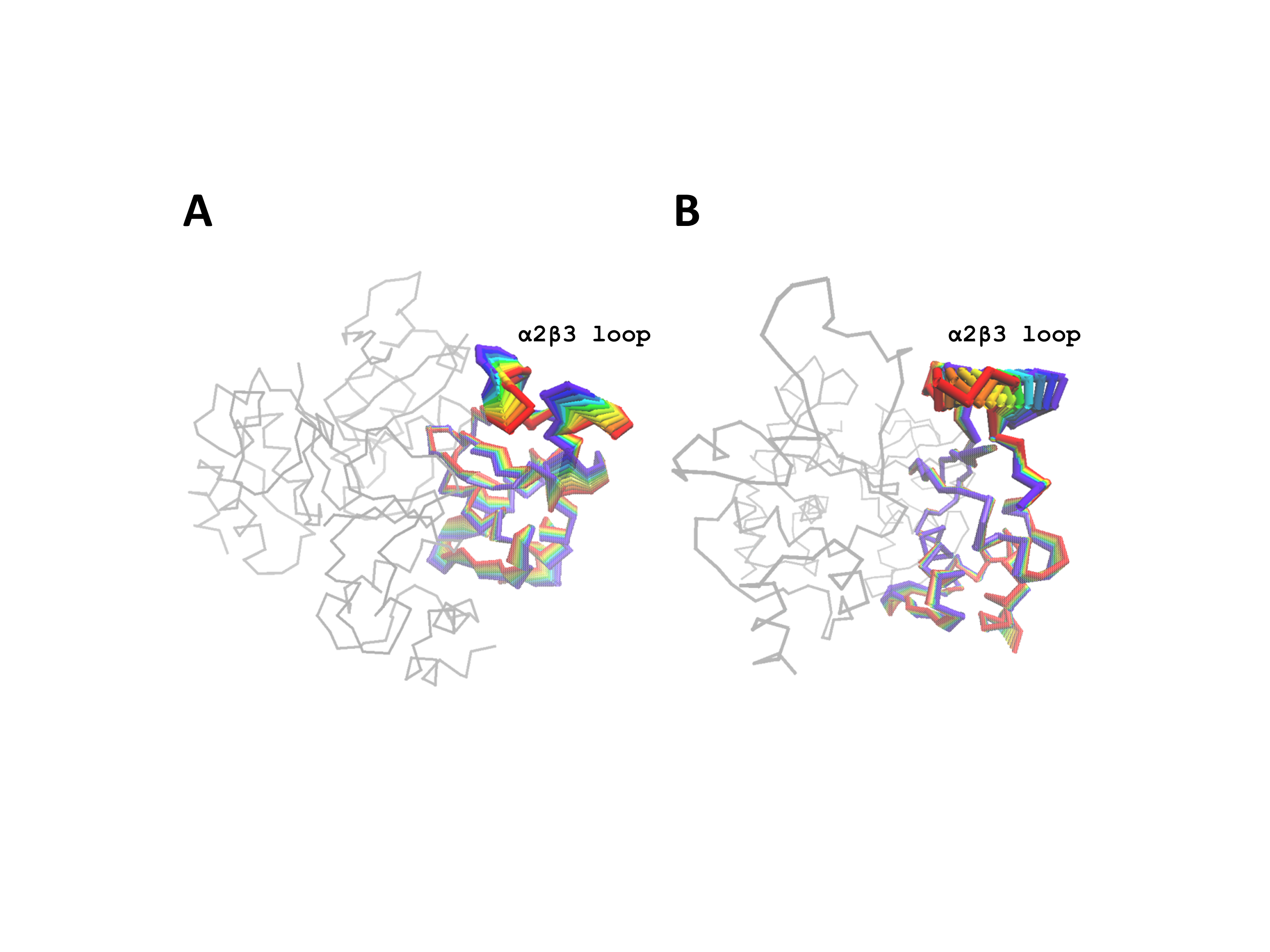

Supplement: S8 Fig — The differentiated motions along the first eigenvector are represented in a film-like fashion. Large movements are displayed by the α2-β3 loop region. An arbitrary color scale (from violet to red) is used to represent the movement. For clarity, the motion of a single chain within the tetramers is shown. (TIF) [file pone.0126808.s008.tif]
